# Outcomes of segmentectomy versus lobectomy in adults with non-cystic fibrosis bronchiectasis

**DOI:** 10.36416/1806-3756/e20240301

**Published:** 2024-12-17

**Authors:** José de Sá Moraes, Isabele Alves Chirichela, Alessandro Wasum Mariani, Ricardo Mingarini Terra, Paulo Manuel Pêgo Fernandes

**Affiliations:** 1. Departamento de Cirurgia Torácica, Instituto do Coracao, Hospital das Clínicas HCFMUSP, Faculdade de Medicina, Universidade de São Paulo, São Paulo, SP, BR.

**Keywords:** Bronchiectasis, Thoracic surgery, Disease-free survival

## Abstract

**Objective:**

Surgical resection remains the gold standard treatment for bronchiectasis in patients who present with hemoptysis or suppuration, as well as in those who do not respond to clinical treatment. We sought to investigate the efficacy of sublobar resection (segmentectomy) and compare it with that of lobar resection (lobectomy) in patients with non-cystic fibrosis bronchiectasis.

**Methods:**

Patients undergoing lobectomy or segmentectomy between 2019 and 2023 were included in the study. We analyzed intraoperative complications and postoperative outcomes, including length of hospital stay, length of ICU stay, and disease recurrence.

**Results:**

There was no significant difference between the lobectomy and segmentectomy groups regarding the occurrence of intraoperative complications such as bleeding > 1000 ml, cardiogenic shock, and ventilatory instability (p > 0.999). However, the frequency of complications was significantly lower in the segmentectomy group than in the lobectomy group (p = 0.016). Hospital stays were longer in the lobectomy group than in the segmentectomy group (16 days vs. 5 days; p = 0.027), as were ICU stays (7 days vs. 1 day; p = 0.006). There was no significant difference between the lobectomy and segmentectomy groups regarding the recurrence rate (p = 0.541).

**Conclusions:**

Early identification of bronchiectasis patients who are candidates for surgical resection is essential because those who are identified as such early on are candidates for parenchyma-sparing resections, which are similar to lobar resections in terms of disease control and lead to shorter hospital stays and better postoperative outcomes.

## INTRODUCTION

Infectious lung diseases are a heterogeneous group of diseases including community-acquired pneumonia and rare fungal infections.^1^ In a small portion of cases, irreversible changes in the lung architecture can lead to chronic conditions. Common features include irreversible dilatation and distortion of the bronchial tree, the causes of which are many.^2^ The incidence of bronchiectasis in adults ranges from 3.7 cases/100,000 population to 52 cases/100,000 population.^2^

Pulmonary tuberculosis is the main cause of postinfectious bronchiectasis. Pulmonary tuberculosis is a public health problem mainly in developing countries, despite public health policies aimed at mitigating it. Most patients with pulmonary tuberculosis develop pulmonary sequelae such as cavitation, which occurs in approximately 40%.^3^ ^,^ ^4^

Surgical resection for bronchiectasis consists of removing permanently damaged lung areas into which antibiotic penetration is poor, serving as a reservoir for bacteria and fungi and thus leading to recurrent infections.^5^

Anatomical lung resection (lobectomy) is the procedure of choice for the surgical management of bronchiectasis; however, sublobar resections have been used as parenchyma-sparing procedures in cancer patients.^6^ Sublobar resections have been validated in multicenter randomized studies in the field of thoracic oncology, with survival rates and perioperative complications similar to those of lobar resections.^7^ However, there is a lack of studies investigating the efficacy and safety of sublobar resections in comparison with lobar resections in patients with infectious lung diseases. 

Although the two procedures are theoretically the same from a technical point of view, they can be very different in terms of how they are performed and the postoperative complications that can occur in cases of cancer or inflammatory disease, the rate of postoperative complications being high in patients with inflammatory or infectious diseases.^8^

Given the lack of studies examining sublobar resections in patients with bronchiectasis, the objective of the present study was to estimate the efficacy of sublobar resection by calculating disease-free survival and complication rates, as well as by analyzing postoperative outcomes such as duration of chest tube drainage, length of hospital stay, and length of ICU stay, in patients with non-cystic fibrosis (CF) bronchiectasis undergoing sublobar or lobar resection. 

## METHODS

This was a single-center retrospective study of prospective data from the Research Electronic Data Capture (REDCap; Vanderbilt University, Nashville, TN, USA) database maintained by the Thoracic Surgery Department of the University of São Paulo School of Medicine *Hospital das Clínicas* Heart Institute, located in the city of São Paulo, Brazil. All data are anonymous and were collected anonymously, thus ensuring the confidentiality of patient data. 

Patients > 18 years of age with non-CF bronchiectasis undergoing sublobar resection between 2019 and 2023 for the surgical treatment of complications or as an adjunct to clinical treatment were compared with those undergoing lobar resection for symptom control. All of the patients included in the present study underwent chest CT, the reported findings being consistent with bronchiectasis. Data on the cause of bronchiectasis were collected from patient medical records, including symptoms (suppuration and hemoptysis), a history of pulmonary tuberculosis confirmed by diagnostic tests, and occupational exposure. Patients with CF bronchiectasis were excluded. Other exclusion criteria were as follows: patients with missing data on the type of resection performed and those without outpatient follow-up. 

All data were entered into a Microsoft Excel spreadsheet for statistical analysis. Categorical variables were expressed as absolute numbers and percentages. Numerical variables with normal distribution were expressed as mean and standard deviation, whereas those with asymmetric distribution were expressed as median and interquartile range. The demographic data were compared by means of the chi-square test, the t-test, or the Mann-Whitney test, depending on the type of variable. Values of p < 0.05 were considered significant. 

The study was approved by the University of São Paulo School of Medicine *Hospital das Clínicas* Research Ethics Committee (Protocol no. 6.750.919/CAAE 33365720.2.0000.0068) and was conducted in accordance with the Declaration of Helsinki. 

## RESULTS

Of a total of 65 patients undergoing surgical resection for bronchiectasis between 2019 and 2023, 27 were excluded because of missing data on the type of resection performed or lack of outpatient follow-up. Therefore, the study sample consisted of 38 patients in the 19- to 71-year age bracket (mean age, 47.77 ± 13.78 years). Most were female and White (61% and 76%, respectively). Table 1 shows the general characteristics of the study population. As can be seen in the table, there was a significant difference between the patients who underwent lobar resection (the lobectomy group) and those who underwent sublobar resection (the segmentectomy group) in terms of percent predicted FEV_1_ (p= 0.003). Pulmonary tuberculosis sequelae were the main cause of bronchiectasis in both groups (47% in both groups), and hemoptysis followed by suppuration was the most common reason for surgery (in 21% of those in the lobectomy group and in 12% of those in the segmentectomy group). 

**Table 1:** General characteristics of the study sample, by type of surgical treatment. ^a^

	Total sample	Type of surgical treatment		p
		Lobectomy	Segmentectomy	
	(N = 38)	(n = 25)	(n = 13)	
Age, years	47.77 ± 13.78	47.85 ± 14.06	47.62 ± 13.78	0.951
VEF_1_, % predicted	73.32 ± 23.17	65.32 ± 23.71	88.69 ± 11.89	0.003
Operative time	292.89 ± 87.96	304.60 ± 91.90	270.38 ± 78.30	0.281
				
Sex				0.136
Female	23 (61%)	13 (52%)	10 (77%)	
Male	15 (39%)	12 (48%)	3 (23%)	
Race				0.793
Asian	1 (2.6%)	1 (4.0%)	0 (0%)	
White	29 (76%)	18 (72%)	11 (85%)	
Black	8 (21%)	6 (24%)	2 (15%)	
Etiology of bronchiectasis				0.185
Aspergillosis	7 (18%)	3 (12%)	4 (31%)	
Galvanization	1 (2.6%)	0 (0%)	1 (7.7%)	
Histiocytosis	1 (2.6%)	0 (0%)	1 (7.7%)	
Repeat infection	9 (24%)	7 (28%)	2 (15%)	
Tuberculosis	18 (47%)	13 (52%)	5 (38%)	
Tuberculosis/aspergillosis	2 (5.3%)	2 (8.0%)	0 (0%)	
Indication for surgery				0.857
Fungus ball	3 (7.9%)	1 (4.0%)	2 (15%)	
Hemoptysis + fungus ball	1 (2.6%)	1 (4.0%)	0 (0%)	
				
Hemoptysis + suppuration	1 (2.6%)	1 (4.0%)	0 (0%)	
Hemoptysis	21 (55%)	14 (56%)	7 (54%)	
Suppuration	12 (32%)	8 (32%)	4 (31%)	

a Data expressed as mean ± SD or n (%).

Whether a given patient is a candidate for lobar or sublobar resection depends on the degree of lung parenchyma involvement. In the group of patients who underwent segmentectomy, 2 underwent wedge resection and 11 underwent anatomical resections, the most common being right upper lobe anterior segmentectomy (in 4 patients), followed by left upper lobe trisegmentectomy. In the group of patients who underwent lobectomy, right upper lobectomy was the most common procedure (performed in 15 patients), followed by left upper lobectomy (in 9). 

As can be seen in Table 2, there was no significant difference between the lobectomy and segmentectomy groups regarding the rate of bleeding (p = 0.385) or the occurrence of intraoperative complications such as bleeding > 1000 ml, cardiogenic shock, and ventilatory instability (p > 0.999). 

**Table 2:** Association between bleeding and the type of surgical treatment.

Surgical treatment	Total sample	Bleeding		p
		No	Yes	
	(N = 38)	(n = 31)	(n = 7)	
Lobectomy	25 (66%)	19 (61%)	6 (86%)	0.385
Segmentectomy	13 (34%)	12 (39%)	1 (14%)	

As can be seen in Table 3, there was a significant association between the type of treatment and surgical complications (p = 0.016). The frequency of complications was significantly lower among those who underwent segmentectomy than among those who underwent lobectomy (15% vs. 56%; p = 0.016). Complications included prolonged air leak and clinical signs and symptoms such as fever, hypotension, postoperative pain, empyema, and hemothorax. Although there were no significant differences between the lobectomy and segmentectomy groups regarding the rate of bleeding or the occurrence of intraoperative complications, there was a marginal association between the type of treatment and the need for reoperation (Table 4). 

**Table 3:** Association between complications and the type of surgical treatment.

Surgical treatment	Total sample	Complications		p
		No	Yes	
	(N = 38)	(n = 22)	(n = 16)	
Lobectomy	25 (66%)	11 (44%)	14 (56%)	0.016
Segmentectomy	13 (34%)	11 (85%)	2 (15%)	

**Table 4:** Association between reoperation and the type of surgical treatment.

		Reoperation		
	Total sample (N = 38)	No (n = 31)	Yes (n = 7)	Value of p
Surgical treatment				0.072
Lobectomy	25 (66%)	18 (58%)	7 (100%)	
Segmentectomy	13 (34%)	13 (42%)	0 (0%)	

**Figure 1:**
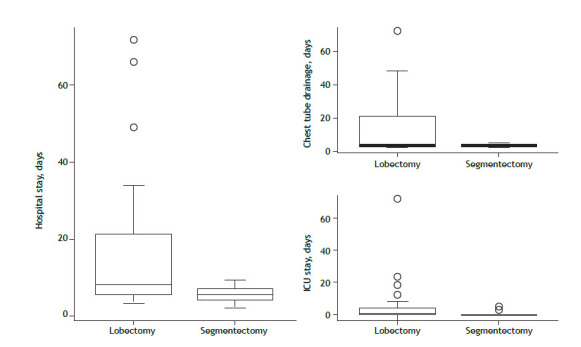
Length of hospital stay, ICU stay, and chest tube drainage in patients undergoing lobectomy or segmentectomy for the surgical treatment of non-cystic fibrosis bronchiectasis.

The recurrence rate is an important factor to consider when analyzing parenchyma-sparing resections. As can be seen in Table 5, there was no significant difference between the lobectomy and segmentectomy groups regarding the recurrence rate (p = 0.541). As can be seen in Figure 1, the patients who underwent lobar resections had longer ICU and hospital stays. 

**Table 5:** Association between recurrence and the type of surgical treatment.

		Recurrence		
	Total sample (N = 35)	No (n = 32)	Yes (n = 3)	Value of p
Surgical treatment				0.541
Lobectomy	22 (63%)	21 (66%)	1 (33%)	
Segmentectomy	13 (37%)	11 (34%)	2 (67%)	

## DISCUSSION

Chronic lung infection, particularly pulmonary tuberculosis, is an important area of expertise for thoracic surgeons, especially those in developing countries. In patients with bronchiectasis and symptoms such as hemoptysis and suppuration, resection of the affected area is the gold standard treatment.^4^ However, robust prospective and retrospective studies on this topic are currently scarce. 

In the present single-center study, we investigated the efficacy of sublobar resection (segmentectomy) in comparison with that of lobar resection (lobectomy) in patients undergoing surgical treatment for non-CF bronchiectasis. We analyzed perioperative complications and disease-free survival, both of which are important when analyzing parenchyma-sparing resections. 

In the group of patients undergoing lobar resection, pulmonary function tests were significantly lower. ^(^ ^8^ In addition, operative time was longer in the lobectomy group than in the segmentectomy group, although the difference was not significant. This means that pulmonary involvement was more severe in the patients who underwent lobar resection, the procedure presenting greater technical difficulty. 

Pulmonary tuberculosis was the leading cause of bronchiectasis (in nearly half of the patients in the present study), a finding that is consistent with the literature. ^(^ ^2^ Hemoptysis and suppuration were the most common reasons for surgery, occurring in approximately 90% of the study population. Hemoptysis has been reported to account for approximately 40% of all surgical indications. ^(^ ^9^ Approximately twice as many patients in the lobectomy group vs. the segmentectomy group had bleeding > 1,000 ml and complications during the surgical procedure, including bleeding, air fistula, hemodynamic instability, and ventilatory instability.

There was a marginal association between lobectomy and the need for reoperation, the main causes being empyema and hemothorax. However, there was a significant association between lobectomy and the occurrence of perioperative complications. In addition, the patients in the lobectomy group had longer hospital stays, longer ICU stays, and longer chest tube drainage. 

The results of the present study are similar to those of a study conducted by Yang et al., ^(^ ^3^ who reported that sublobectomy and lobectomy were both effective in the surgical treatment of cavitary pulmonary tuberculosis. However, the sublobectomy group had fewer intraoperative blood losses, shorter stays, and fewer perioperative complications than did the lobectomy group (p < 0.05). ^(^ ^3^

With regard to the disease recurrence rate in the present study, there was no significant difference between the lobectomy and segmentectomy groups. 

Patients undergoing lung resection tend to have pulmonary involvement that is more severe and, consequently, more severe clinical and functional deterioration, all of which are closely related to intraoperative and perioperative outcomes. Patients undergoing lobar resection tend to have more complications, including a higher bleeding rate. 

Early identification of bronchiectasis patients who are candidates for surgical resection is essential because those who are identified as such early on are candidates for parenchyma-sparing resections, which are similar to lobar resections in terms of disease control and lead to shorter hospital stays and better postoperative outcomes. 
